# Identification of methicillin-resistant *Staphylococcus aureus* within the Nation’s Veterans Affairs Medical Centers using natural language processing

**DOI:** 10.1186/1472-6947-12-34

**Published:** 2012-07-11

**Authors:** Makoto Jones, Scott L DuVall, Joshua Spuhl, Matthew H Samore, Christopher Nielson, Michael Rubin

**Affiliations:** 1 VA Salt Lake City Health Care System, Salt Lake City, UT, USA; 2 Department of Internal Medicine, University of Utah, Salt Lake City, UT, USA; 3 VA Reno Medical Center, Reno, NV, USA; 4 University of Nevada, Reno, NV, USA

## Abstract

**Background:**

Accurate information is needed to direct healthcare systems’ efforts to control methicillin-resistant *Staphylococcus aureus* (MRSA). Assembling complete and correct microbiology data is vital to understanding and addressing the multiple drug-resistant organisms in our hospitals.

**Methods:**

Herein, we describe a system that securely gathers microbiology data from the Department of Veterans Affairs (VA) network of databases. Using natural language processing methods, we applied an information extraction process to extract organisms and susceptibilities from the free-text data. We then validated the extraction against independently derived electronic data and expert annotation.

**Results:**

We estimate that the collected microbiology data are 98.5% complete and that methicillin-resistant *Staphylococcus aureus* was extracted accurately 99.7% of the time.

**Conclusions:**

Applying natural language processing methods to microbiology records appears to be a promising way to extract accurate and useful nosocomial pathogen surveillance data. Both scientific inquiry and the data’s reliability will be dependent on the surveillance system’s capability to compare from multiple sources and circumvent systematic error. The dataset constructed and methods used for this investigation could contribute to a comprehensive infectious disease surveillance system or other pressing needs.

## Background

There is a pressing need for timely, reliable, and generalizable information to guide infection control efforts directed against methicillin-resistant *Staphylococcus aureus* (MRSA) within hospitals. This microorganism frequently causes abscesses, bloodstream infections, post-surgical infections, and sometimes deaths; estimates from existing research and census data suggest that 17,000 attributable deaths occurred in 2008 [1,2]. With the objective of reducing MRSA transmission in hospitals, the Department of Veterans Affairs (VA) implemented the National MRSA Prevention Initiative in October 2007 [3]. The program included VA-wide MRSA testing upon admission to, discharge from, and transfers between acute care wards; rules for contact precautions; hand hygiene; a change in culture to one of shared responsibility; and new reporting systems [4]. The VA Inpatient Evaluation Center (IPEC) gathered data to evaluate this program by employing coordinators at each facility to review MRSA results. The current mode of data collection could be augmented and made more efficient with detailed electronic microbiology data. These electronic data could also be used for algorithmic surveillance, which has the advantage of reliability over time and place [5].

Microbiology data are increasingly collected electronically throughout the United States and could eventually provide a powerful means of infectious disease surveillance, but the synthesis and utilization of databases across large networks remains a daunting endeavour both in and out of the VA. Barriers include differing data models [6], messaging strategies, and security issues [7]. The VA medical centers have had an electronic medical record system for over 20 years. This includes, but is not limited to, microbiology data maintained at 152 hospitals currently active worldwide. These data are siloed at each hospital, complicating the process of compiling and integrating enterprise-wide data. Re-engineering the system to capture standardized, structured data will eventually be performed, but was a prohibitively large undertaking at the time. Hence, our objective was to evaluate methods that permitted rapid extraction and validation of these microbiology data.

The VA stores patient-level microbiology and most other types of data in a hierarchical health information system called the Veterans Health Information Systems and Technology Architecture (VistA). VistA uses a programming language and database called MUMPS (Massachusetts General Hospital Utility Multi-Programming System). Although all VA medical centers use the same software programs, they may have distinct naming conventions and some variation in data structure [8]. This allows flexibility but also permits redundancy and idiosyncrasies to creep into the data. There has been some consolidation of VistA instances among medical centers, but most continue to maintain their own VistA system. Because a core system integrating microbiology data across VistA systems was not otherwise available, we utilized an available system developed by VA Patient Care Services (PCS).

**Figure 1:**
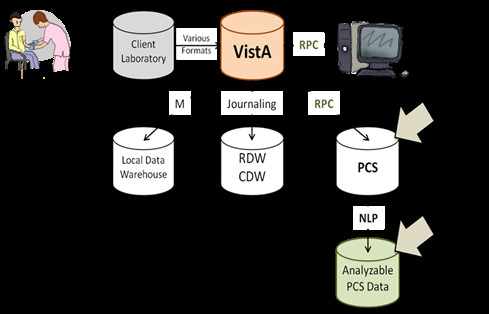
**Data Network Diagram.** RPC - remote procedure call. CPRS - Computerized Patient Record System. RDW- regional data warehouse. CDW-corporate data warehouse. PCS – VA Patient Care Services. NLP- natural language processing. Laboratory data are generated when tests on patient samples are processed in laboratories and entered into VistA. From there, they may be accessed through CPRS and PCS through RPCs. They may also be extracted through other processes (journaling and M). Because PCS microbiology data are free-text, information must be extracted into an analyzable form. Figure courtesy of Kiyoshi Jones.

The PCS system used Medical Domain Objects, an approach similar to the process that retrieves records during the course of clinical care (see Figure 1). Healthcare providers normally access data using the CPRS (Computerized Patient Record System) graphical user interface. The CPRS interacts with the core MUMPS databases through a number of established remote procedure calls (RPCs) that execute patient data objects (objects that assemble data to form reports or components of reports for visual display). The process for using a RPC is identical at all medical centers and is highly reliable. VistaWeb, developed a number of years ago to access VistA data off-site, also uses RPCs. VistaWeb is in daily use nationwide and enables off-site access to medical records [9].

The PCS system uses the VistaWeb interface to execute RPCs at each medical center and then uploads the data to a Structured Query Language (SQL) relational database in a secure VA data center. The VA login and network security processes are based upon the same approaches required for providers system-wide. Because the PCS system uses existing, reliable data processes, it requires no independent maintenance and can be run as a background process.

The separation of RPCs and patient data objects can have a valuable role in maintaining data validity. Data extracted through RPCs represent a coupling of both VistA data and the patient data object, which means that information extracted this way retains context from the patient data object that would not otherwise be present. RPCs invoke universal commands to local patient data objects that incorporate the meta-data necessary to make VistA data intelligible to providers. If data structures or reporting formats change within an implementation of VistA then local programmers must also update patient data objects so that RPCs continue to retrieve appropriate data to display for healthcare workers. Thus, the data that healthcare workers see, interpret, and report errors about are the same data extracted through the PCS process. When raw VistA data are pulled into a central database, often by different teams than those that built the local patient data objects, the data must be carefully evaluated for changes in structure and semantics. This is critical because microbiology records often contain multiple tests and a hierarchical structure of cultures, microorganisms, and susceptibilities.

Direct data retrieval can retain native data structure, but is unable to retrieve ‘misplaced’ data. This is because 1) not all of the complex aspects of microbiology reports were anticipated when the data model was developed; 2) microbiology reports from client laboratories may have formats incompatible with the data model; and 3) MUMPS has only one data type, so there are no data type checks. As a result, data may be systematically or sporadically entered in the wrong place in VistA, but still be correctly represented to the provider from RPCs used by CPRS.

The methodology that we investigated retrieved VA-wide, patient-level data in the same format used by health care providers. These records are in a semi-structured, free-text form that is as “human-readable” as an official microbiology report. Even though standardized, individual fields were lost by using this format, the record can be inspected visually to interpret its meaning. But as the data were already assembled and could be updated daily, they represented a valuable resource in need of formal validation.

## Methods

### Description of information extraction process

Microbiology records from the beginning of 1990 through the end of 2009 were collected from all VistA systems for this task. This study was approved by the Research Review Committee of the VA Salt Lake City Health Care System and Institutional Review Board of the University of Utah.

Before information extraction, we removed MRSA surveillance tests from the microbiology data using a filter on cultured sample and specimen types. To develop this filter, 11,596 unique sample and specimen types were reviewed manually by one of the authors (MJ) and annotated as to whether they were consistent with a MRSA surveillance test from the anterior nares. Such tests are performed to identify silent carriers and not clinical disease. Their report formats are substantially different from that of routine culture and susceptibility tests and were therefore removed for later extraction in analyses of MRSA transmission. While the reports gathered by PCS provided microbiology data from VA sites nationwide, the free-text format of these reports necessitated further processing. To identify the organisms mentioned in these reports along with their antibiotic susceptibilities, we employed a set of natural language processing (NLP) techniques for information extraction. The extraction creates a formal representation of concepts in the text that can be used in computer algorithms.

**Figure 2:**
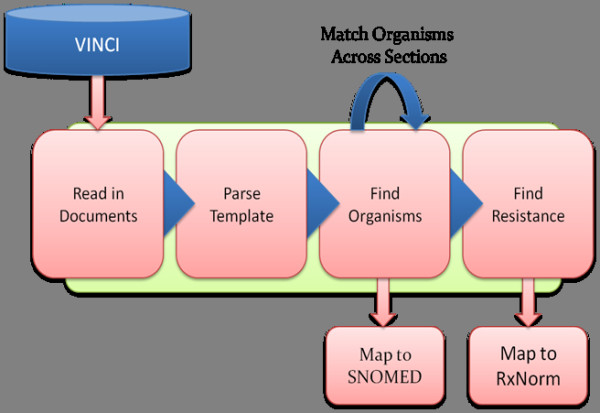
**Information Extraction Strategy.** VINCI -VA Informatics and Computing Infrastructure. SNOMED – Systematized Nomenclature of Medicine. RxNorm – a standardized nomenclature of clinical drugs developed by the National Library of Medicine.

The NLP system was developed in the Apache Unstructured Information Management Architecture (UIMA), an open-source framework that provides a consistent data model and interface for handling annotations and meta-data associated with unstructured data such as text [10]. The UIMA supports development of multi-stage applications where individual processes are used together in sequence to achieve a final result. This pipeline of processes is cumulative, with each step using information added from previous steps to perform more complex tasks. While the most common approach to NLP is grammatical – parsing sentence structure and assigning semantic meaning based on the syntactic role each word plays – this requires the text to be “natural language.” The microbiology reports were not laid out in normal sentences and paragraphs, but followed regular templates with concept-value pairs and table structures. Therefore, we took advantage of the native structure to determine the semantic meaning and infer relationships between concepts. Our approach was to craft specific rules to handle specific structures. General rules were applied when specific rules were not. The pipeline was composed of four general tasks: section identification, organism detection, susceptibility detection, and MRSA inference (see Figure 2).

**Figure 3:**
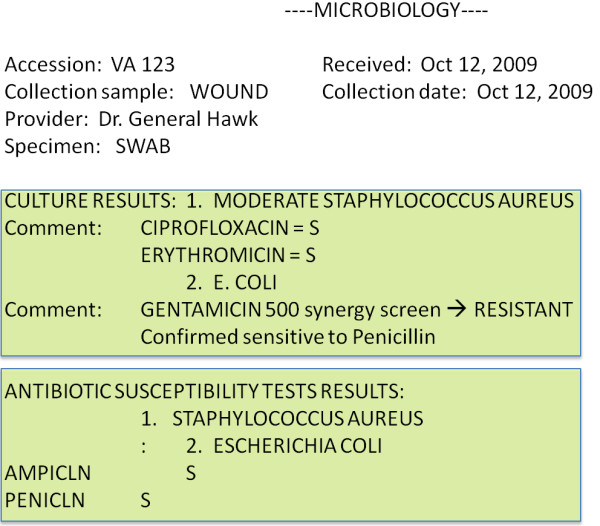
**Sample of a Microbiology Report.** A typical microbiology report is represented here. Metadata about the specimen taken are described first. There are two sections here: ‘Culture Results’ and ‘Antibiotic Susceptibility Test Results.’ The organisms listed in both are linked by numbers because the same genus and species may be isolated more than once. Susceptibilities may appear in the ‘Culture Results’ section with the organism name, in the same section in a ‘comments’ subsection as depicted above, or in a matrix in the ‘Antibiotic Susceptibility Test Results’ section as depicted above.

1. Section identification: Although the microbiology reports were free-text, the semi-structured format of the reports allowed for the decomposition of the full document into sections. The sections, constructed by patient data objects, contained consistent information across VA sites. Specific rules were crafted to take advantage of these sections and to identify which types of concepts of interest may be found within. Using this approach, the system extracted the templated meta-data contained in each report. Some of these data, such as accession number, site and specimen of the sample, and dates and timestamps were already available in structured data.

2. Organism detection: Lines of text that contained organisms were found using rules that leveraged the structural cues of common formats, such as numbered lists and indented lines in the Culture Results section and in tables in the Antibiotic Susceptibility Test Results section. Once these lines were found, the name of the organism (either a descriptor like cocci or bacilli, a genus like *Staphyloccocus*, or a full genus and species like *Staphyloccocus aureus*) was split from descriptors such as quantity or concentration, descriptions of resistance (MRSA), and results of other identifying tests (Gram negative, alpha-hemolytic). We found that the variation in the ways that organisms were recorded was much greater than the variation of the descriptors. Thus, we created patterns and prepared a list of terms that represented quantity, concentration, resistance, etc. Search terms were developed in an iterative process using the training reports. We started by analyzing just a few reports and building the initial terms/rules. In each iteration, we added new reports from the training set and performed a detailed failure analysis. The results of the failure analysis informed us which new terms/rules needed to be added. The text remaining after each of the descriptors was identified became the name of the organism. The organism names were subsequently mapped to SNOMED-CT.

3. Susceptibility detection: We detected antibiotic susceptibility tests by determining the rules used in the susceptibilities section of each microbiology record. Drawing from our process of iterative development, we knew that susceptibility-associated terms would most often be found with their organism names in free-text comments in the Culture Results section and in tables in the Antibiotic Susceptibility Results section (see Figure 3). For example, when a list of organisms was presented, the susceptibility was either recorded in the list (e.g., “1. Staph. Aureus - Methicillin Resistant”) or as a comment between list elements (e.g.,“1. Staph. Aureus Comment: This organism is methicillin resistant. 2. E. Coli”). Susceptibility most commonly occurred in tables at the end of the report where organisms were listed as column headers and antibiotics as row headers. The intersecting cell at each row and column determined the susceptibility of that organism to that antibiotic. Susceptibility terms were mapped to logical values of resistant, susceptible, or indeterminate for computation. 

4. MRSA inference: If *Staphyloccocus aureus* (*S. aureus*) was one of the organisms detected in the microbiology report, the detected susceptibilities were used to determine whether the culture was positive for methicillin resistance.

Records were processed on the VA Informatics and Computing Infrastructure (VINCI), a high-performance computing environment that provides researchers a secure, central location for data access and application development.

### Reference comparisons

We used reference data sets from two sources: routine MUMPS extractions of VistA data into local data warehouses and manual annotation of raw PCS data. The first set was extracted and manipulated by data managers at their native sites just as they normally would and was used as a standard for assessment of completeness and correctness. The second set was only used for an assessment of the correctness of PCS data. We concluded that the manual review of raw PCS data was an acceptable alternative to accessing each record through VistAWeb because a sample of 142 records from across the VA verified that raw-text records from PCS data were substantively, character-by-character concordant with the text of the same records accessed through VistAWeb. All character mismatches were attributed to clinically irrelevant data, such as laboratory certification numbers that sometimes change during the time between when the data are pulled and when VistAWeb is accessed. Having data derived from alternative, independent data extraction methodologies was critical to ensuring a valid estimate of data completeness and the correctness of our information extraction [11]. An ideal reference would have been from a health information system that records and stores all patient information accurately, immediately, and unambiguously [12,13]. No such system exists because of human, logistical, and technological limitations, but the electronic health record, accessed through CPRS, is the accessible source of information closest to our ideal. Observations recorded through the CPRS system allow for review and correction of ambiguities or inaccuracies. However, CPRS is only accessible manually, so we made comparisons with other extractions from VistA. To evaluate the PCS microbiology set, we have assessed the completeness, concordance, and correctness of MRSA data.

### Evaluation of completeness

A complete microbiology data set would be ideal, but it is more important that the data not be subject to selection bias while still providing adequate sensitivity for outbreak detection. Estimates of completeness facilitate assessments of the data along these criteria. We compared PCS data to independently-derived data sets and evaluated completeness by linking microbiology tests in each set and measuring the concordance of their presence. Unfortunately, the same set of unique keys were not available in both sets, so we used the microbiology accession number (a nearly unique identifier for microbiology tests when coupled with the location and patient identifiers) and collection time to perform the linkage. Logistic and practical considerations precluded use of a VA-wide sample of independently pulled data. To compensate, we also estimated the lower bounds of completeness by analyzing meta-data that were sequential in nature contained in the PCS tables.

We used patient data from the VA Salt Lake City Health Care System (VA SLC HCS) (data from 2005–2008) and the Veterans Integrated Service Network (VISN) 4 (a network of ten VA hospitals, data from 1999–2006) data warehouses for comparisons. VISN 4 microbiology organism and susceptibility had been coded independently by the VISN 4 staff. Because data warehouse and PCS data were retrieved by entirely independent processes and methodologies, concordance provided a reliable indication that these data accurately reproduced the original VistA database.

The other method to estimate completeness relied on the standard practice of sequentially ordering microbiology accession numbers at each VA site. Microbiology accession numbers are made up of a short string identifier (for example “bc” for blood culture, “micro” for microbiology, or even a short-name for a send-out laboratory), a date component, and a sequential number. We encountered two types of sequential numbering strategies: one incremented accession numbers with any type of culture and the other separated incremental counters for each culture type. For simplicity, when separate incremental counters were used, we used the dominant sample type for our analysis. Since there is a wide variation in sample naming and numbering conventions between medical centers, it was necessary to manually identify conventions at each site. Gaps in sequence were identified between cultures. When a sequence gap was found, this was taken to be the maximum number of cultures potentially missing from the database. Unfortunately, sequence gaps occur for reasons other than missing patient data (gaps might be filled with non-patient samples from laboratory animals and hospital surfaces), so this method only produces a lower bound estimate. This analysis was performed for each data warehouse data set, as well as, a sample of ten randomly sampled VA hospitals.

### Evaluation of the extraction and inferencing of MRSA

The original PCS report text was used as a reference to evaluate the accuracy of the NLP system. Ten thousand randomly selected microbiology documents containing the strings “staph”, “coag”, “mrsa”, “orsa”, “oxacillin”, or “aureus” were queued for manual, expert review. Clinical nurses with experience reading and interpreting microbiology reports were trained to use a secure web-based application to annotate the presence of *S. aureus*; whether methicillin resistance was present, absent, or not documented; and whether the record was an MRSA surveillance test. Reviewers were blind to the NLP results, but were free to consult with each other and with the authors. Synonyms for S. *aureus* included accepted abbreviations, MSSA, MRSA, and coagulase-positive S. *aureus*. Our standard for the definition of methicillin resistance was based on Clinical Laboratory and Standards Institute guidelines [14]. Historically, not all VA sites have listed oxacillin, cefoxitin, penicillin binding protein 2A or mecA testing on microbiology reports, so resistance to cefazolin, cephalothin, nafcillin, or simply a statement of methicillin resistance were permitted as well. Screening studies from the data set aside for expert review were identified by reviewers, but removed during evaluation of information extraction accuracy. Separate training and test sets were generated from this sample by randomly selecting half to go into each set.

Microbiology data from VISN 4 and the Salt Lake City Health Care System, which had already been annotated, were also used for comparison. Half of the records were randomly chosen to become part of the training set (all of Salt Lake City data were used for training), while the other half became part of the test set. Sensitivity and specificity were calculated separately when PCS data were compared with expert-reviewed and electronic data sets. For purposes of comparison, the absence of mention was treated the same as mention of absence during expert annotation and in the extracted data set. Confidence intervals were calculated assuming normal distributions. Because of the enormity of the set, errors during expert annotation due to fatigue and repetition, and the use of other algorithms to identify MRSA in comparison data sets, we anticipated the need to re-review and sometimes over-rule records that were preliminarily categorized as discordant. We also planned to review 200 concordant records (50 at the *S. aureus* and MRSA levels for each modality) anticipating that true concordance was > =98%.

## Results

### Description of the data

Using PCS extraction methods to build the data set, microbiology data from January 1, 1990 to December 31, 2009 were available for analysis. The data set included 33,024,796 unique records from 128 VistA sites representing 152 currently active acute care hospitals and 170 total hospitals during the entire time frame. It was apparent that microbiology report formats changed over the years and varied between centers, but the core content of organism and susceptibilities was constant.

### Completeness of the data

We estimated PCS data completeness through comparison with data warehouse (DW) patient data, which were available from eleven VA sites with average yearly admissions of 2,842 (range from 356 to 7080). Matches on microbiology accession numbers were found to represent 98.5% [95% CI 98.5-98.6%] of the whole. As concordance was high and served as a lower bound for report completeness, further investigation of the discordant set was not pursued.

By analyzing microbiology accession sequences, we estimated the lower bounds PCS data completeness for the same sample of eleven hospitals. Five hospitals were found to be missing more than five percent of accession numbers, but we found that these sequence gaps were almost entirely attributable to quality control and other non-patient sampling. An additional ten hospitals were randomly selected to examine sequence gaps. Four of ten additional randomly selected hospitals demonstrated missing accession numbers of greater than 5%, but their data warehouse data were not available for comparison. We also attempted to assess temporal gaps and combinations of sequence and temporal gaps to assess completeness. However, because we observed the presence of long gaps, particularly at nights and on weekends, we did not pursue the identification of single or small numbers of missing cultures with this method.

### Accuracy of the data

As mentioned, MRSA screens were removed from the microbiology data by means of a string-searching algorithm we developed. This algorithm identified MRSA surveillance screens among our 10,000 expert-annotated charts with a 99.4% sensitivity and 97.9% specificity.

 The successful extraction of *S. aureus* and methicillin resistance from PCS data was assessed by comparison to DW data and an expert annotated random sample from the PCS data. Four thousand and two records were identified as screens in the expert reviewed data set; they were removed before the information extraction analysis. We included half of the remaining 5,998 records in our training set from a random nationwide sample that were manually reviewed by experts; 5,967 records were documented for both *S. aureus* status and methicillin resistance from MUMPS extracted data from ten VISN 4 hospitals (see Table 1). In addition, 53,627 microbiology records from VA SLC HCS that documented the presence or absence of MRSA were included. When determining the categorization of *S. aureus* in the training set, the sensitivity was 99.6%, specificity 99.9%, and PPV 99.9%. We found a sensitivity of 99.9%, a positive predictive value (PPV) of 99.7%, and a specificity of 99.9%, with respect to the correct assignment of methicillin resistance.

**Table 1:** Information extraction accuracy

**Training Set**						
	*Records Reviewed : 62,500 (53,627 records from SLC only annotated for MRSA)*					
			Sensitivity	Specificity	PPV	NPV
	*Staphylococcus aureus*		99.6 (4026/4044)	99.9 (4828/4829)	99.9 (4026/4027)	99.6 (4828/4846)
		Methicillin Resistance	99.9 (2789/2790)	99.9 (59701/59710)	99.7 (2786/2795)	99.9 (59701/59705)
**Validation Set**						
	*Electronic Records Reviewed: 5,927*					
			Sensitivity	Specificity	PPV	NPV
	*Staphylococcus aureus*		100 (2739/2739)	99.9 (3185/3188)	99.9 (2739/2742)	100 (3185/3185)
		Methicillin Resistance	100 (1460/1460)	99.9 (4465/4467)	99.9 (1460/1462)	100 (4465/4465)
	*Expert Reviewed Records: 3,092*					
			Sensitivity	Specificity	PPV	NPV
	*Staphylococcus aureus*		98.3 (1348/1372)	99.7 (1714/1720)	99.6 (1348/1354)	98.6 (1714/1738)
		Methicillin Resistance	99.2 (703/710)	99.4 (2368/2383)	97.9 (703/718)	99.8 (2368/2374)

PPV - positive predictive value, NPV - negative predictive value depicts the accuracy of the extraction process on the training set (both electronic and expert-reviewed data sets combined), as well as on the validation set (reported separately). Both numbers and percentages are supplied.

We then made comparisons using a second set of 5,927 electronic and 3,092 expert-reviewed records. Sensitivity, specificity, and PPV for identification of *S. aureus* were estimated at 100%, 99.9%, and 99.9% compared to electronic data and 98.3%, 99.7%, and 99.6% compared to expert-reviewed data (see Table 1).

Susceptibilities were analyzed in a similar fashion. Among records that successfully identified *S. aureus*, we found that there were 57 discordant records in the electronic set and 53 in the expert-reviewed set. Discordant records were re-reviewed manually (by MJ) on both the manually and electronically derived test sets. Discordant records from the expert-reviewed set usually contained simple errors likely due to reviewer fatigue, while discordant records from independently extracted electronic data often used the term ‘coagulase-positive *Staphylococcus*’ to identify *S. aureus* (it must be noted that we were unable to elucidate how the classification had been made initially in the electronic data set). Forty-seven records were resolved in favor of our extraction in the electronic data set, while 18 records were resolved in favor of our extraction in the expert-reviewed data set. After this re-review, there was a concordance of 99.8% with the electronic and 98.9% with the expert-reviewed data sets. Sensitivity, specificity, and PPV were estimated at 100%, 99.9%, and 99.9% compared to electronic data and 99.2%, 99.4%, and 97.9% compared to expert-reviewed data. Results are summarized in Table 1. Of the 200 concordant records re-reviewed for concordance, all records were found concordant.

## Discussion

We demonstrated the successful compilation and extraction of a large and very valuable data set using available data and tools. Data were securely and completely extracted from hospitals all over the nation. Experts were involved during the development of natural language processing tools and inference algorithms. In accordance with established frameworks to evaluate data quality [11,12,15], we assessed the extraction of free-text microbiology with respect to accuracy. Validation was accomplished through the expert annotation of human-readable documents. Although the process of generating a corpus of expert-annotated notes was time intensive, our approach allowed us to utilize structural information embedded in the microbiology reports to ensure proper interpretation.

Our assessment of this large database was limited not only by the absence of a valid reference standard, but also by the amount of accessible electronic data available for comparison. A random sample from more facilities would have improved our analysis of completeness, but logistically was not feasible. Instead, we augmented our study by analyzing microbiology accession number sequence gaps at ten VA hospitals and demonstrated that roughly half of these sites demonstrated significant gaps. However, when we examined sites where DW data were available, we found that in roughly half of the hospitals these gaps were largely due to non-patient samples. We cannot conclude from these data that we have complete data from the unsampled VA sites; however, our results suggest that many VA sites are complete and that some, if not most, of the accession number gaps observed do not represent patient data loss. It appears from our analysis that missing data are unlikely to be systematically related to elements analyzed in clinical studies.

The analysis of correctness was assisted by determining that PCS records were text copies of CPRS reports. We reviewed a large sample of microbiology data from all available VA medical centers to capture the variability that may have occurred in the microbiology reports over time and place. Even with 10,000 random samples, our sample size from each of the individual VA sites was not large, particularly from small VA facilities. Thus, we were unable to report VA site-specific estimates of accuracy. The high accuracy scores we have reported are reassuring that the natural language processing methods are robust and will serve well in the future, as well as, against other organisms.

Our data warehouse comparisons highlighted the fact that not all routine data warehouse pulls for research are validated with respect to completeness and concordance. This deficiency needs to be addressed because systematic biases can be easily introduced when using data derived from or are processed by electronic means. In large, multi-centered studies where data are compared that originate from separate databases, this is of particular concern. VINCI, an entity supporting informatics and research within the VA, is dedicated to developing data sets for research through transparent and reproducible methods and validation against independently extracted data.

Operationally, this data set could contribute to comprehensive surveillance systems for infectious diseases. Although time-consuming, the practice of comparing expert-annotations of the electronic medical record version of the microbiology report with extracted data could improve the reliability of data. We presume that variation in report formats might be even greater when combining data from disparate non-VA health systems. Because clinical data are used to anticipate costs and influence healthcare practice, the extra time and money spent to validate data before analysis are offset whenever we prevent actions based on false conclusions [16].

## Conclusions

Very large, incongruent data sets can be efficiently integrated in ways that also make them epidemiologically sound. Here, we delivered MRSA surveillance data from over a twenty-year time span in one of the largest healthcare systems in the United States. Our methodology can inform groups that find themselves in similar situations – where needed data are not easily accessible, come from multiple sources, and may require groups with complementary skills to work together. We utilized available options and pulled together a cost-effective, reproducible result. We emphasize the importance of using clinical experts. We discuss the use of NLP and decision rules, but show how performing evaluations at each step (from the data pull, to the NLP extraction of concepts, to determining relationships between concepts, to classifying reports) is crucial when handling very large and potentially unpredictable data sets.

## Competing interests

None of the authors have received reimbursements, fees, funding, or salary from an organization that may gain or lose financially from this publication. There are no outstanding patents to which any of the authors have any interest. We have no other competing interests. All of the authors do have affiliations with the Department of Veterans Affairs.

## Authors’ contributions

MJ was involved with data collection, acted as a domain consultant for organism extraction, developed the analysis, and drafted the manuscript. SLD was involved with developing and performing information extraction, participated in the analysis, drafted the information extraction methods of the manuscript, and helped with the writing of the manuscript in general. JS was involved with data collection, manipulation, analysis, and helped draft the manuscript. MHS was responsible for the study’s conception, participated in the design, and helped draft the manuscript. CN performed data extractions from VistAs, participated in data modeling, drafted the data extraction methods, and helped with the draft of the manuscript in general. MR participated in the study’s conception, design, analysis, and manuscript drafting. All authors read and approved the final manuscript.

## Pre-publication history

The pre-publication history for this paper can be accessed here:

http://www.biomedcentral.com/1472-6947/12/34/prepub
